# 

**Published:** 1891-09-19

**Authors:** 


PRICE ONE PENNY.

No. 260, Vol. X.—September 19th, 1891.

[Registered at the General Post Office as a Newspaper for transmission in the United Kingdom and Abroad.]

WITH EXTRA NURSING SUPPLEMENT.

Per Annum, 6s. 6d.; post free.

Monthly Parts, 6d.; post free 8d.

Ditto per Annum, 8s., post free.

### THE HOSPITAL

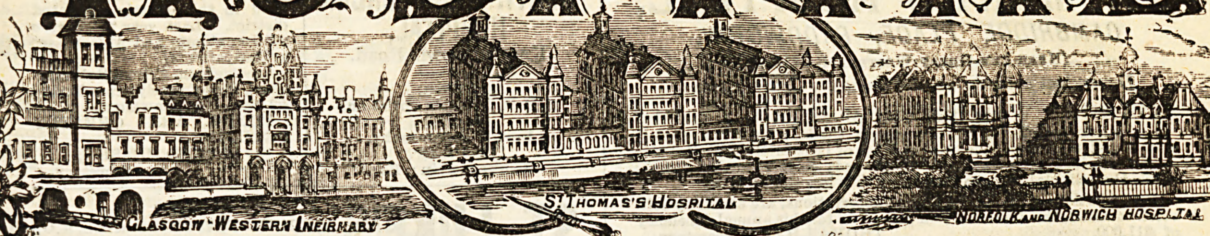

A WEEKLY JOURNAL OF
Science, Medicine, Nursing and Philanthropy.

### SATURDAY, SEPTEMBER 19th, 1891.

**Midnight Oil or Midnight Sleep?** · · · · · · 287

**Passing Topics.**—Shall Nurses Cease to be Laywomen?—II. · · · · · · · · · · · · 288

**The Doctor's Armchair.**—Corn and Husks · · · · 289

**"Medicus Nascitur, non Fit"** · · · · · · 290

**Everybody's Page** · · · · · · · · · · 291

**Editor's Letter-box.**—"How Doctors are Made."—The Dangers of Chloroform · · · · · · · · 291

**Notes and News** · · · · · · · · · · 292

**General Charities** · · · · · · · · · · 293

**Scraps and Gleanings** · · · · · · · · · 293

**Annotations.**—Doctors' Training in Lunacy—A Battle All but Won—Village Life · · · · · · · · 294

**The Practitioner's Mirror and Retrospect**—

Surgery.—Some Points in the Diagnosis of Hip Disease in Children—Diseases of the Fingers (*concluded*). G.—The Skin, &c. · · · · · · · · · · 295

Therapeutics.—Somnal · · · · · · · · 296

New Drugs, Appliances, and Things Medical.—British Medical Association (*continued* —"Frame Food' Company 297

**The Student of Medicine.**—Appointments—Vacancies—Scholarships and Prizes—Pass Lists · · · · · · 298

### EXTRA SUPPLEMENT—THE NURSING MIRROR.

**En Passant.**—Rural Nurses—Unsatisfactory Nurses—Short Items—Another Society—Marriage—Alexandria—Nurses' Food—Housekeeping Details · · · · · · cxliii

**A Fever Hospital** (By One who has Been There) · · cxliv

**The Princess of Wales and the Nurses** · · · cxliv

**A Few Words to Nurses** · · · · · · · · cxlv

**For Reading to the Sick**—The Lesson we Teach · · cxlv

**Our Indian Letter** · · · · · · · · · · cxlvi

**Everybody's Opinion.**—Drunken Nurses—The R.B.N.A. Outdoor Uniform, cxlvi; Corrections · · · · · cxlvii

**Appointments** · · · · · · · · · · · cxlvii

**Presentation** · · · · · · · · · · · cxlvii

**Notes and Queries** · · · · · · · · · · cxlvii

**Wants and Workers** · · · · · · · · · cxlvii

**Amusements and Relaxation** · · · · · · · cxlvii

**Off Duty.**—Content to Follow · · · · · · cxlviii

